# Medical Jurisprudence

**Published:** 1837-10

**Authors:** 


### MEDICAL JURISPRUDENCE.

### *Cases of Infanticide, with Remarks.*

[THE great importance of the subject, and the interesting nature of the details communicated, are, we conceive, sufficient excuse for the extent and minuteness with which we have reported the following cases. They form an appropriate appendix to the Article on *Infanticide* in our last Number.]

CASE I. *Reported by* DR. SCHRECK, *of Grünstadt.*

On the 7th November, 1833, the body of a new-born female child was found concealed in a house at Grünstadt, under suspicious circumstances. The medico-legal examination took place on the 8th, and the following appearances were met with:—The body measured twenty inches in length, and weighed about five pounds and a half. The umbilical cord, which had been torn across the middle, was of about the average length. The head was well covered with short hair, and the nails were perfect, especially those of the fingers. The skin of the lower and fore part of the neck was discoloured, as if from violent handling; and on the right side were five small black spots apparently resulting from the pressure of fingers. On cutting through the skin at this part, the discoloration was found to extend throughout all the layers. The most important marks of violence were, however, in the face. The cheeks were torn through from the angles of the mouth to the ears. The lower jaw was fractured into three pieces; and the cornua of the os hyoides were torn from the soft parts and forced backwards. Blood was found extravasated beneath the scalp, as also on the surface of the brain.

On removing the parietes of the chest, the lungs were seen lying at the back of that cavity, and only covering a small part of the pericardium laterally. They were of a pinkish red colour, but their anterior surface was darker than the posterior. The lungs, when placed on water, with the heart and thymus attached, floated almost on a level with the surface of the liquid. The lungs, when detached, weighed about two ounces and a half. On cutting them into pieces, there was no evident crepitation from the escape of air. These pieces readily floated on water. The foramen ovale and ductus arteriosus were open and pervious, as in the fœtal state. The abdomen presented nothing unusual; but meconium was found in the whole tract of the colon.

The medical opinion given from the result of this examination led to the trial of the mother for the alleged murder of her child, before the Assize Court of Zweibrücken, on the 6th March, 1834.

The medical evidence adduced on the occasion was to the following effect:

1. The child was born alive.
2. The child was born capable of living.
3. It had died a violent death.
4. That, very probably, the marks of violence observed on its body might not have been wilfully inflicted, but produced by the violent efforts of the mother, in endeavouring to accomplish her own delivery.

The first three points, the witnesses alleged, admitted of an easy exposition.

1. That the child was born *alive* was proved not only by the state of the lungs, but by the appearance of the marks of violence about the face and neck. The fact of the ecchymosis extending through the whole of the substance of the skin was a manifest proof that, at the time these marks were produced, a complete circulation of the blood was going on in the body. That respiration had not been carried on long was proved by the imperfect dilatation of the lungs and the perviousness of the foramen ovale and ductus arteriosus.

2. That the child was born *capable of living* might be determined from its general characters. There was no defect in its organization. At the same time, the incomplete state of the cartilages of the ears and of the nails of the toes, as well as the fact of its weight not exceeding five pounds and a half, were circumstances which rendered it probable that it was not quite mature.

3. The marks of *violence* found on the body were undoubtedly the cause of death. The laceration of the cheeks, the fractures of the lower jaw, and the displacement of the os hyoides, proved that very great force must have been used. The displacement of the larynx would have interrupted the respiratory process; and, taking the whole of the injuries together, they were such as must speedily have destroyed life.

4. In relation to this point, the chief medical witness stated in court that he had not met with any instance in the records of medical jurisprudence where a new-born child had been wilfully destroyed in a manner similar to this. It was rare that the marks of criminal violence on the bodies of children were of so extensive and severe a nature.

The violence he observed, may be supposed to have resulted from a forcible pulling of the head during delivery, and the compression which the shoulders and hips might at the same time have experienced would account for the discolorations found on those parts of the body. It was easy to imagine that a female, wholly unacquainted with the process, might, during a protraction of her sufferings, be driven to such a state of frenzy and excitement as to induce her to tear her offspring with violence from her. The accused in this case may have made such attempts, and thus have unintentionally produced the marks of fatal violence. In relation to the protraction of delivery, it was admitted that the pelvis of the mother was of the natural dimensions; and that, had the position of the child been such as is commonly observed, the delivery must have taken place without difficulty, especially since the body was small. But the witness argued that the delivery might have been protracted by a malposition of the child; that the head might have protruded, while the shoulders remained fixed. He then proceeded to assume that the accused might have introduced her fingers into the child's mouth, and in her efforts at self-delivery have accidentally caused the lacerations and fractures.

The court having left it to the jury to say whether the prisoner had wilfully destroyed her child, a verdict was returned to the effect that she had killed it not wilfully, but through imprudence and neglect.

[*Remarks.* In this case, the fact of the child having *lived* appears to us to have been clearly established, directly by the experiments on the lungs, and indirectly by the vital characters of the marks of violence on the body. These marks might, however, have presented precisely similar characters, even had the child not breathed, so that the evidence derived from the circumstance of the whole of the layers of the skin being penetrated by blood would have been of little value, had it not been corroborated by the use of the hydrostatic test

2. The second question which the witnesses had to answer upon the trial is founded on a principle peculiar to the laws of certain parts of the continent; namely, whether the child was born *capable of living*, (*lebensfähig, viable.*) This capability of living is usually determined by noting whether a child be born with all its organs perfect and healthy, and at a due period of gestation. To those who are acquainted with the jurisprudence of this country it must appear extraordinary that such a preliminary proof should be required in a question of child-murder. The capability of living in this child is fully established by the proofs of development described in the examination.

3. That the child was destroyed by violence is sufficiently clear from the medico-legal report.

4. With regard to the origin of this violence, we are inclined to think, that Dr. Schreck's explanation of the circumstances, although highly ingenious, and certainly within the range of *possibility*, was a little too constrained. It is probable, however, that other moral evidence was adduced to show that the mother had no intention of destroying her child, and that it was unlikely from circumstances she had done this wilfully or with criminal intention. In her case every fact was favorable to a rapid delivery; it was assumed that the process might have become protracted from malposition of the child after protrusion of the head. But it is not often we find, in a well-formed pelvis, and under a head-presentation, that such violent and extensive injuries are required to be inflicted, in order to expedite delivery.

The jury virtually acquitted the prisoner of infanticide, and found her guilty of involuntary homicide, a crime which would correspond in our law to one description of manslaughter.]

CASE II. *Reported by* PROFESSOR VON SIEBOLD, *of Göttingen.*

[We have been induced to select this case from the singular resemblance which it bears in its details to the preceding, and from the very different conclusion to which the medical examiners came respecting the origin of the marks of violence on the child.]

The prisoner, a well-formed female, was secretly delivered on the night of the 23d July, 1834. Suspicion having been strongly excited against her, the body of the child underwent a medico-legal examination. It was of the male sex, and had apparently reached the full period. The umbilical cord had been forcibly torn through. There was a laceration, extending through the right cheek from the corner of the mouth to the ear. Both of the rami of the lower jaw were broken, and the right ramus was dislocated. The skin of the neck was swollen and of a greenish blue colour. A considerable laceration extended from the mouth downwards to behind the first bone of the sternum: by this the larynx and trachea had been forced away from the soft structures, and the œsophagus had been torn through. Blood was extravasated in all the parts around. Coagula of blood were found beneath the skin of the scalp. Both of the parietal bones were fractured, the right especially; its central portion presenting several loose osseous fragments. The contents of the cranium, thorax, and abdomen were free from all abnormal appearances. The experiments performed with the lungs clearly showed that the child had breathed.

The statement of the prisoner was, that she felt the motions of the child the day previous to her delivery; but that it did not seem to be alive at its birth. She ascribed the marks of violence on its body to the efforts which she had made to deliver herself. When she felt the head issuing she stated that she introduced her hand into its mouth and pulled violently, by which something seemed to give way. To this she attributed the laceration of the cheek and neck, and the fractures of the jaw. These efforts at self-delivery were made while she was in the *recumbent posture.* She did not observe that the child breathed or cried at any time. After her delivery she tore asunder the umbilical cord, and then placed the dead body in the straw beneath the bed.

From the result of the inspection the following report was made:

1. The child was mature and born capable of living.

2. It lived before and during birth.

This inference was derived from the absence of putrefaction, which showed that the child had recently lived, and from the experiments performed on the lungs.

3. It is also highly probable that the child had lived and breathed after its birth.

This presumption was derived from the roundness and width of the chest; from the dilatation of the lungs, the pericardium being covered by the right lung; from the crepitation, escape of air on pressure, and absolute weight of these organs; and, lastly, from their complete buoyancy in water, as well with the heart and thymus attached as when they were divided into numerous small pieces.

In relation to the objection that the child might have breathed during delivery, it was contended that, as the woman had already borne a child, and the labour, according to her own statement, was rapid, it was improbable that any delay should have taken place in the birth of the body after the passage of the head. At the same time, it was admitted that no *positive* opinion could be given on this point. On the other hand, the life of the child after its birth was rendered probable by the evident discharge of meconium, and by the ecchymosis and other characters of the violence existing on the body. That the child had been wilfully and violently destroyed was, in the opinion of the examiners, proved by the serious injuries to the head and neck, and by the laceration of the untied umbilical cord. It is contended that the mother could not, for the purpose stated by her, so have applied her hand as to break and dislocate the jaw, lacerate the cheek and œsophagus, and, at the same time, force the trachea and larynx down towards the thorax. The employment of the hand for the purpose of self-delivery was not likely to have produced such extensive and varied effects. The direction of the marks of violence on the neck was such as to lead to the belief that, in producing it, the child's head would rather have been forced backwards into the passage than drawn outwards. Again, had the hand been introduced into the mouth, in the manner and for the purpose stated, some violence would most probably have been done to the upper jaw and palate: but there were no traces of violence to these parts. Finally, they declared that the child had died in consequence of the injuries which it had sustained.

The advocate who defended the accused raised some objections to the opinions expressed by the examiners; and the court accordingly referred to the Medical Faculty of G— for answers to the following questions:

1. Whether respiration might not have been set up prior to birth by the prisoner, in her alleged efforts at self-delivery, accidentally conveying air to the mouth of the child?

2. Whether the prisoner could have produced the injuries to the jaw and face, in the manner stated by her, during delivery; whether, in this case, there ought or ought not to have been marks of violence on the upper jaw and palate.

The faculty answered the first question by stating that the child, setting aside the efforts alleged to have been made by the mother, might have breathed so soon as its head was born. The introduction of her hand into the child's mouth might also have facilitated the establishment of respiration.

In answering the second question, they stated it as their opinion that, from the depositions, the delivery must have been throughout natural, easy, and rapid; that such violent efforts at self-delivery were not from the circumstances likely to have been required; that if, as the prisoner affirmed, she was in the recumbent position, she could not so have employed her hands, as to produce the severe injuries on the child, or have seen the process, so as to know in what direction the head and the face lay. It was wholly improbable, that the injuries should have been produced in the manner described by her. The violence to the lower jaw, if caused by the attempts at self-delivery, would most probably have been accompanied by corresponding violence to the upper jaw. Admitting that these extensive injuries had been produced by the prisoner during the birth of her child, it is certain that she must have employed a much greater degree of violence than any female in her situation, for the *mere purpose of aiding delivery*, was likely to have employed.

[*Remarks.* This case forms an excellent contrast to the preceding. In the first, the whole of the talent of the examiner was put in requisition in order to make out a defence for the prisoner on bare *possibilities.* In the second, the prisoner herself raised this defence. We do not see that there existed any want of humanity in those who conducted the last investigation; but we recognize in the solid reasons which they adduce in support of their opinions respecting the *wilful* and *probably criminal* origin of the violence, a stern desire to uphold justice without favour or partiality. This is, in our view, as it should be: a medical jurist is not called upon to judge of a crime or of its punishment, but to determine upon the consistency and correctness of the medical evidence required to support it. If a Court of Law were to admit every plausible hypothesis or possible conjecture that a clever witness could put forward on trials for murder, and were to act upon such admission, it is certain that but few convictions would take place. The framing of laws for the repression of crime would indeed become a vain and fruitless task, if a jury were not permitted to take a common-sense view of the medical and other facts proved.

In this case, the faculty, in their decision, made every reasonable allowance for the condition of the woman, and for the efforts which she might have made during delivery: but her own account of the labour was inconsistent with the idea that such efforts should have been required, or, if required, that they could possibly have been attended with the whole of the marks of violence found on the body of her child. Besides, we must regard it as rather the character of guilt than of innocence that she should have afterwards concealed the child. The circumstantiality with which she described the progress of her labour, as also the manner in which, according to her own view, each injury or mark of violence was to be accounted for—the remaining, according to her own account, the whole of the time in a recumbent position,—are points which tell very little in favour of her innocence.]

*Henke's Zeitschrift für die Staatsarzneikunde.* 1836.

